# Multiple Myeloma Complicating Breast Cancer During Treatment: A Case Report

**DOI:** 10.3390/curroncol33080473

**Published:** 2026-08-10

**Authors:** Lijing Guo, Zhengshuo Jin, Yuehua Huang

**Affiliations:** 1 Department of General Surgery, Beijing Tsinghua Changgung Hospital, School of Clinical Medicine, Tsinghua Medicine, Tsinghua University, Beijing 102218, China; glja02305@btch.edu.cn; 2 Department of Hematology, Beijing Tsinghua Changgung Hospital, School of Clinical Medicine, Tsinghua Medicine, Tsinghua University, Beijing 102218, China; jzsa03981@btch.edu.cn

**Keywords:** multiple myeloma, breast cancer, HER2-positive, chemotherapy, multiple primary malignant neoplasms

## Abstract

**Research Background:** Breast cancer is a malignant tumor that threatens women’s health. Among breast cancer patients, about 4.2% to 17% may suffer from multiple primary malignant neoplasms. Cases of patients suffering from both breast cancer and multiple myeloma are relatively rare in clinical practice. This paper reports one case of a patient who developed multiple myeloma during the treatment of breast cancer. **Clinical Data:** The patient was a 56-year-old female, admitted to hospital due to “fever for 4 days, one year after the treatment of left breast cancer”. The patient was diagnosed with left breast cancer (ypT1N1M0, HER2-overexpressing subtype) approximately one year prior to presentation. Following diagnosis, the patient was treated sequentially with TcbHP and THP chemotherapy regimens, and subsequently received local radiotherapy. The patient developed fever four days prior to admission. Further laboratory examinations revealed pancytopenia, positive IgA-λ-type monoclonal immunoglobulin by immunofixation electrophoresis, and myeloma cells accounting for 11% in bone marrow smears. Bone marrow immunophenotyping indicated abnormal plasma cells, and pathological results of bone marrow biopsy were consistent with multiple myeloma. The final diagnosis was breast cancer complicated with multiple myeloma. After confirmed diagnosis, the patient was transferred to another hospital for targeted treatment of multiple myeloma and has been receiving continuous treatment there up to the time of follow-up. **Conclusions:** For patients with breast cancer, if they develop bone pain, anemia, hypercalcemia or renal dysfunction that cannot be explained by tumor metastasis or conventional complications after surgery or during follow-up, clinicians should be alert to the possibility of second primary tumors.

## Simple Summary

Breast cancer is a leading malignant tumor threatening women’s health. The co-occurrence of breast cancer and multiple myeloma is clinically rare. We herein report a 56-year-old female patient with left breast invasive ductal carcinoma who developed secondary IgA-λ multiple myeloma one year after breast cancer treatment, presenting with fever and pancytopenia. She received targeted myeloma treatment and remains under continuous therapy. This case reminds clinicians to screen for secondary hematological malignancies in breast cancer patients with unexplained atypical symptoms during follow-up.

## 1. Background

Female breast cancer represents the most frequently diagnosed malignancy among women globally. According to GLOBOCAN 2022 estimates, approximately 2.3 million new cases were diagnosed worldwide, accounting for nearly one-quarter of all new cancer diagnoses in females, with obvious geographic heterogeneity. High-income regions historically exhibit higher age-standardized incidence rates, while many middle- and low-income countries, including China, have witnessed continuous upward trends over recent decades. Survivors of breast cancer face a slightly elevated long-term risk of second primary malignancies across all populations. Therapy-related myeloid neoplasms are the most commonly reported secondary hematological tumors, whereas metachronous multiple myeloma remains rarely documented globally after multimodal anticancer treatment for breast cancer [1].

Breast cancer survivors carry an elevated long-term risk of second primary malignancies worldwide. Secondary hematological neoplasms represent a well-recognized late complication after cytotoxic chemotherapy and radiotherapy. Population-based registry data demonstrate that therapy-related myelodysplastic syndrome (MDS) and acute myeloid leukemia (AML) are the most prevalent secondary hematologic malignancies among breast cancer patients. The cumulative 10-year risk of treatment-associated MDS/AML ranges approximately from 0.1% to 1.8%, and the risk rises significantly after combined chemotherapy and radiotherapy compared with surgery alone. In contrast, metachronous multiple myeloma (MM) is a much rarer event after breast cancer treatment. Large French and North American cohorts confirm that breast cancer survivors exhibit moderately increased risk for MM, yet absolute incidence remains low at roughly 20.3 per 100,000 person-years. To date, published cases describing secondary MM following neoadjuvant chemo-targeted therapy plus adjuvant radiotherapy for HER2-positive breast cancer remain scarce globally [1,2,3].

This paper reports one case of a patient who developed multiple myeloma during the treatment of breast cancer, analyzing and summarizing the case to provide references for clinical practice, and the details are summarized as follows.

## 2. Case Presentation

### 2.1. General Information

A 56-year-old female presented with a 4-day history of fever approximately one year following curative treatment for left breast carcinoma. One year prior, the patient sought medical attention after accidentally discovering a painless mass in the upper outer quadrant of the left breast. Breast ultrasonography revealed a hypoechoic nodule measuring approximately 2.6 cm × 1.7 cm × 1.8 cm in the areolar area at the 9 o’clock position of the left breast, characterized by ill-defined borders, irregular shape with angular changes, and a small number of punctate blood flow signals inside. Multiple enlarged lymph nodes with thickened cortex sized about 0.9 cm × 0.7 cm were detected in the left axilla. The solid nodule of the left breast was classified as BI-RADS category 5, and multiple enlarged left axillary lymph nodes were highly suspected of metastatic lesions.

Further breast puncture biopsy was performed, and pathological results showed that tumor cells in the punctured breast tissues were arranged in nested and trabecular patterns with varied cell sizes, moderate cellular atypia, and 5 mitoses per 10 high-power fields (HPF). Immunohistochemistry (IHC) results were as follows: P63 (focal positive), Ki67 (70% positive), ER (negative), PR (negative), HER-2 (3+). Combined with all findings, the lesion was diagnosed as invasive breast carcinoma of no special type, grade II, with a total score of 7 points. The final confirmed diagnosis was malignant tumor of the left breast (cT2N1M0, stage IIB, HER2-overexpressing subtype). Routine blood tests at that time showed white blood cell count and hemoglobin level within normal reference ranges, and platelet (PLT) count of 109 × 10^9^/L (reference range: 125−350 × 10^9^/L). Liver and kidney function tests: alanine transaminase 24.4 U/L, aspartate transaminase 23.6 U/L, total bilirubin 7.8 μmol/L, direct bilirubin 4.1 μmol/L, albumin 42.6 g/L, globulin 29.30 g/L, creatinine 55 μmol/L, estimated glomerular filtration rate 103.160, serum calcium 2.24 mmol/L, lactate dehydrogenase 158 U/L. The patient initially received neoadjuvant chemotherapy with the TCbHP regimen: Docetaxel 120 mg (72 mg/m^2^) plus Carboplatin 0.5 g (AUC ≈ 4) administered intravenously on day 1 every 21 days. Targeted therapy was given via subcutaneous pertuzumab and trastuzumab combined injection with an initial dose of 1200 mg and maintenance dose of 600 mg on day 1 every 21 days. The minimum platelet count dropped to 43 × 10^9^/L after treatment, which was diagnosed as grade 3 chemotherapy-induced thrombocytopenia (approximately 10 days after TCbHP administration). Then the chemotherapy regimen was adjusted to a THP regimen: Docetaxel 120 mg (72 mg/m^2^) intravenously on day 1 every 21 days; subcutaneous pertuzumab and trastuzumab combined injection was maintained at 600 mg on day 1 every 21 days. During the third and fourth treatment cycles, pertuzumab injection 420 mg combined with trastuzumab injection 0.4 g was given intravenously on day 1 every 21 days. Partial response (PR) was confirmed after six cycles of chemotherapy.

Half a year ago, the patient underwent left breast-conserving surgery combined with sentinel lymph node biopsy and left axillary lymph node dissection under general anesthesia. Postoperative pathological results revealed invasive breast carcinoma of no special type (grade II, 7 points) in the left breast mass, accompanied by high-grade ductal carcinoma in situ accounting for 90% of the lesion. The tumor measured about 1.2 × 0.8 × 0.7 cm with an invasive focus diameter of 0.1 cm. No vascular tumor thrombus or nerve invasion was observed, and the Miller–Payne (MP) grade after neoadjuvant therapy was grade 4. The status of surgical margins was confirmed by frozen section examination. IHC results of specimen slice No. 11: Calponin (−), P63 (−), P120 (membrane positive), E-Cad (membrane positive), PR (−), CK5/6 (+), AR (90% moderately strongly positive), ER (−), HER-2 (3+), TOPOIIα (30% positive in hot-spot area), Ki67 (70% positive in hot-spot area); Calponin (+) was detected in specimen slice No. 8. Metastatic carcinoma was found in 1 out of 13 dissected axillary lymph nodes.

The patient continued to receive four cycles of targeted HP regimen chemotherapy after surgery. Local radiotherapy including supraclavicular fossa and breast irradiation was initiated two months ago, with a total of 28 fractions. The radiotherapy dose was 50.4 Gy in 28 fractions for 95% planning target volume (PTV) and 59.92 Gy in 28 fractions for tumor bed planning target volume (PTVtb). The platelet count decreased to 36 × 10^9^/L during radiotherapy, and was elevated to 80 × 10^9^/L after symptomatic supportive treatments including blood transfusion and platelet-raising agents.

**Figure 1:**
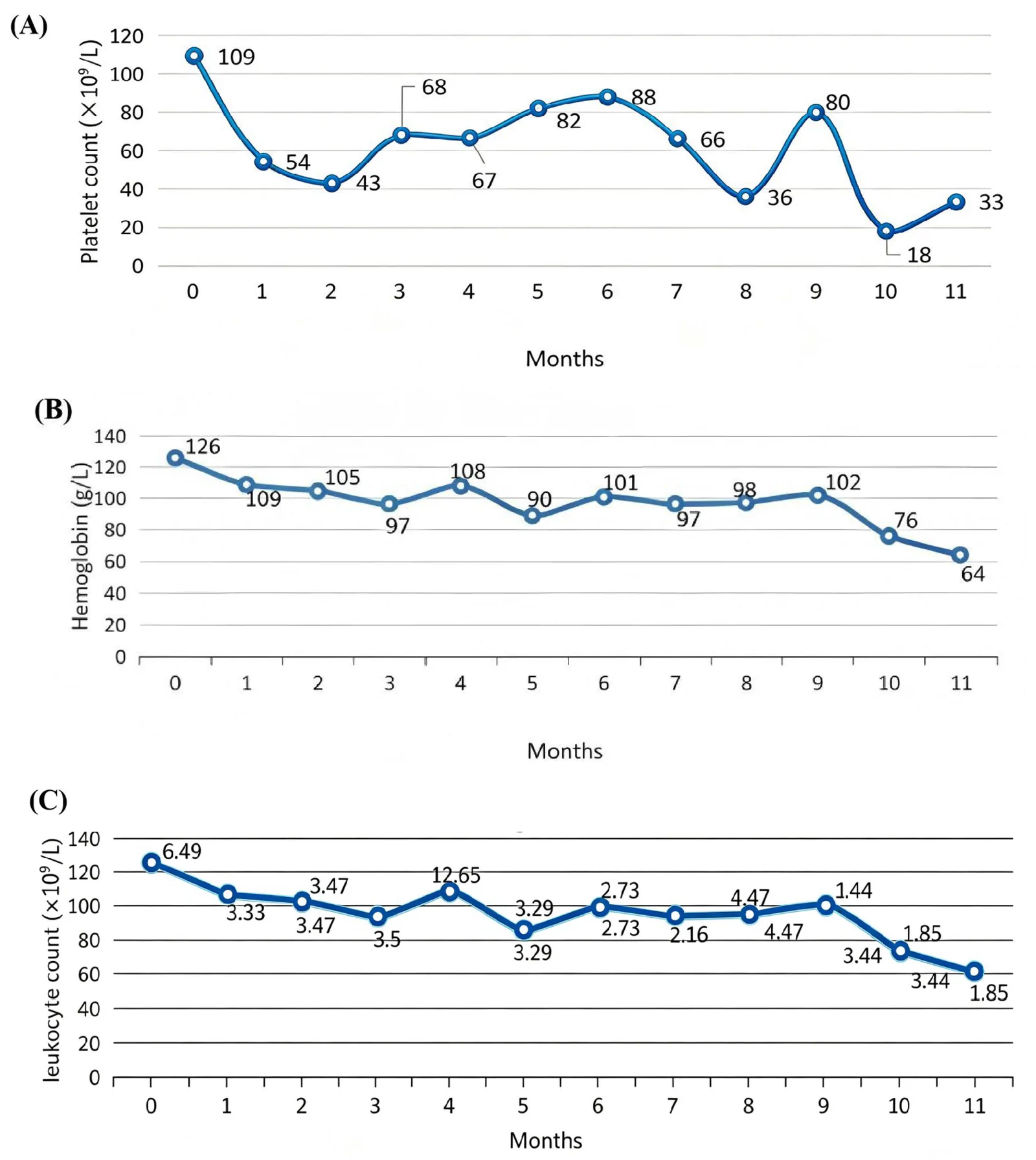
Trend of routine blood test results of the patient during treatment. Serial dynamic changes in hematologic parameters throughout follow-up. (**A**) Platelet count (×10^9^/L); (**B**) hemoglobin concentration (g/L); (**C**) leukocyte count (×10^9^/L).

Regular imaging re-examinations including breast ultrasonography, chest computed tomography (CT) and whole-body bone scan were performed postoperatively, showing no signs of tumor recurrence or distant metastasis. The changing trends of platelet, leukocyte and hemoglobin levels during chemoradiotherapy are shown in Figure 1. The routine blood test results throughout the treatment period are presented in Supplementary Materials: Table S1.

Four days prior to admission, the patient developed fever without obvious inducement with a maximum body temperature of 39.1 °C, accompanied by deepened urine color. Routine urine test revealed full-field red blood cells and white blood cells. The patient did not receive standardized treatment and only took antipyretics and traditional Chinese medicine by herself. Blood tests conducted in our outpatient department showed WBC 3.44 × 10^9^/L, HGB 76 g/L, MCV 94.3 fL and PLT 18 × 10^9^/L, and interleukin-11 was administered to increase platelet count. The patient was hospitalized with a tentative diagnosis of fever of unknown origin.

Since the onset of illness, the patient has suffered poor mental state and appetite, decreased defecation volume, obvious physical weakness, and abnormal urine as mentioned above. The patient has a history of hypertension with the highest blood pressure reaching 170/92 mmHg, which is well controlled by oral valsartan amlodipine tablets. No other underlying diseases such as diabetes mellitus and nephropathy were confirmed.

Physical examination findings: body temperature 39.1 °C, pulse rate 71 beats per minute, respiratory rate 19 breaths per minute, blood pressure 133/78 mmHg with ECOG performance status score of 1 point. The patient was conscious but in poor mental condition with anemic appearance. No jaundice or ecchymosis was found on the skin and mucous membranes, and no superficial lymphadenopathy was palpable. A surgical scar with good healing was seen on the left breast after surgery without residual breast tissue, and no abnormal mass was palpated in the right breast. No obvious abnormalities were found in cardiopulmonary and abdominal physical examinations.

### 2.2. Auxiliary Examinations

Relevant examinations were completed after admission:

1. Reticulocyte percentage 3.12%.
2. Liver and kidney function: ALT 48.0 U/L, AST 19.9 U/L, total bilirubin 16.05 μmol/L, direct bilirubin 8.52 μmol/L, total protein 69.0 g/L, albumin 38.2 g/L, globulin 30.80 g/L, creatinine 69.7 μmol/L, eGFR 85.337, serum calcium 2.06 mmol/L, lactate dehydrogenase 212.4 U/L.
3. Tumor markers: carcinoembryonic antigen (CEA), carbohydrate antigen 125 (CA125) and carbohydrate antigen 15-3 (CA15-3) were all within normal ranges.
4. Imaging examinations: no space-occupying lesions were detected by breast ultrasonography; chest CT showed no signs of metastasis or infection; abdominal and lymph node ultrasonography showed no abnormal manifestations.
5. Other abdominal ultrasonography results were unremarkable.
6. Autoimmune related tests including ENA spectrum, ANA and antiphospholipid antibody spectrum all showed negative results.
7. Routine urine test: occult blood (2+), proteinuria (1+).
8. Infection screening: blood culture yielded extended-spectrum beta-lactamase (ESBL)-positive Escherichia coli (alarm appeared at 11 h and 33 min on day 0); tests for influenza A virus and severe acute respiratory syndrome coronavirus 2 were all negative.

Combined with the patient’s medical history, physical signs and auxiliary examination results, disease progression and bone metastasis of breast cancer were excluded. Chemotherapeutic and radiotherapeutic drugs can damage bone marrow hematopoietic stem cells while killing tumor cells, which may lead to pancytopenia in severe cases. Bone marrow suppression usually occurs 7 to 14 days after treatment, and most blood cell indexes can fully recover to safe levels within about 21 days. The patient’s breast cancer remained stable, and the last course of antitumor treatment ended one month prior to admission, so bone marrow suppression could not explain the abnormal changes in peripheral blood cell counts in this case. In addition, the patient’s blood cell counts failed to recover even after body temperature returned to normal, thus ruling out infection-induced pancytopenia.

**Figure 2:**
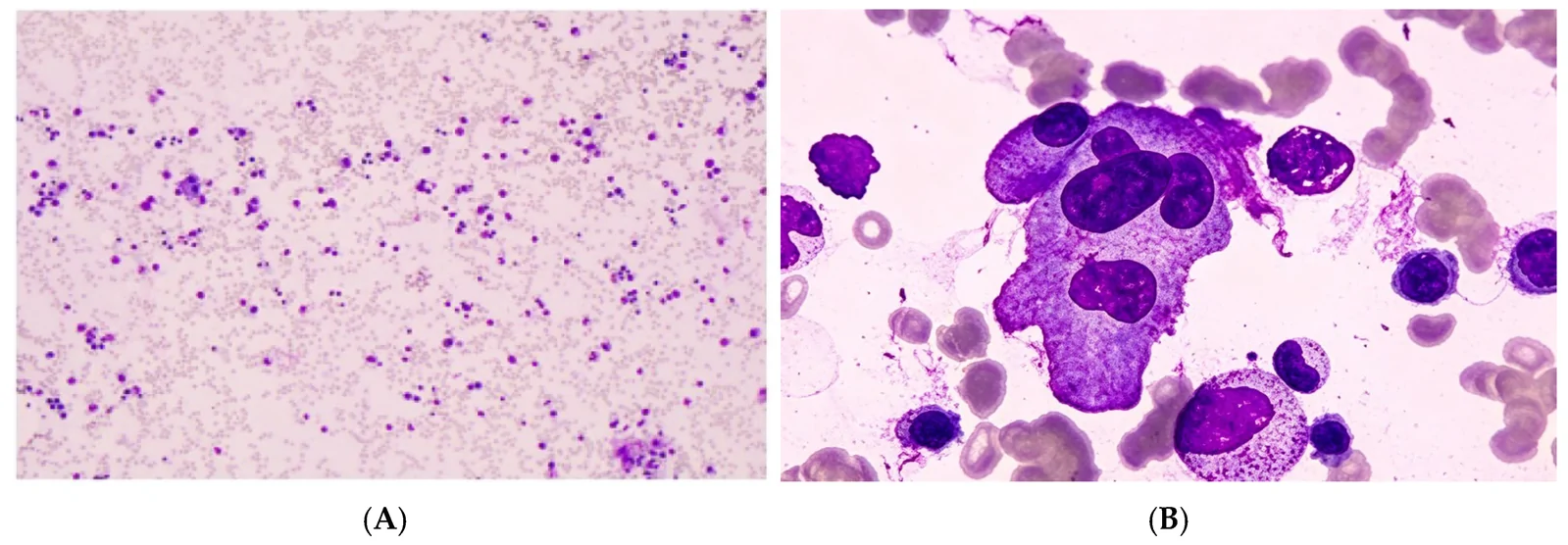
Microscopic manifestations of patient bone marrow smears (Wright–Giemsa staining): (**A**) at 10× magnification, (**B**) at 100× magnification.

After department consultation, hematological system diseases were highly suspected, and further targeted hematological examinations were arranged:

1. Serum vitamin B12 769.0 ng/L, serum folic acid 10.3 μg/L.
2. Serum iron metabolism indexes: serum iron 9 μmol/L, unsaturated iron binding capacity 16.9 μmol/L (normal range: 24.2−70.1), total iron binding capacity 25.90 μmol/L (normal range: 54−77), transferrin saturation 34.75%, serum ferritin 273.9 μg/L (normal range: 13–150).
3. Peripheral blood cell morphology: anisocytosis, leukopenia with toxic granules in partial neutrophil cytoplasm, and rare platelets.
4. Three items of immunoglobulin detection: IgA 7.32 g/L (normal range: 1.0−4.2), IgG 9.9 g/L, IgM 0.42 g/L (normal range: 0.5−2.8).
5. Immunofixation electrophoresis confirmed positive IgA-λ-type monoclonal immunoglobulin with M protein level of 5.17 g/L.
6. Bone marrow examinations: Bone marrow cytology showed hyperactive bone marrow hyperplasia with myeloma cells accounting for 11%, consistent with the bone marrow manifestation of multiple myeloma (shown in Figure 2). Flow cytometry of bone marrow detected 0.49% abnormal plasma cells which expressed CD38, CD138, CD56, CD27, CD81, CD200 and CD28, with restricted cytoplasmic lambda light chain expression.
7. Conventional cytogenetic karyotype: 46, XX.

Bone marrow biopsy pathological results: hyperactive hematopoietic activity was observed in bone tissues and a small amount of bone marrow tissues with all three hematopoietic lineages visible. Proliferative plasma cells accounting for approximately 10% were distributed focally and scattered in the interstitium, presenting mild to moderate atypia with partial immature cells. IHC staining results: CD71 (erythroid lineage-positive), MPO (myeloid lineage-positive), CD42b (megakaryocytic lineage-positive), CD34 (1% positive), CD117 (1% positive), CD3 (scattered T lymphocyte-positive), CD20 (scattered B lymphocyte-positive), CD138 (plasma cell-positive), MUM1 (plasma cell-positive), AE1+AE3 (negative). In situ hybridization results: kappa (focal-positive), lambda (scattered and focal-positive). Special staining results: reticular fiber staining (negative), iron staining (negative).

Integrated clinical manifestations and laboratory findings confirmed the diagnosis of multiple myeloma. The final confirmed diagnoses were listed as follows:

1. Multiple myeloma (IgA-λ type, Durie–Salmon stage III group A);
2. Postoperative status of invasive ductal carcinoma of left breast (ypT1N1M0, HER2-positive, Luminal B subtype);
3. Grade 2 hypertension with high risk stratification.

Upon diagnosis of multiple myeloma, the patient was transferred to another hospital for targeted treatment and continues to receive regular follow-up and therapy at that facility.

## 3. Discussion

Multiple primary malignant neoplasms (MPMNs) refer to the simultaneous or successive occurrence of two or more primary malignant tumors in the same individual, also known as repeated cancers or multiple cancers, which are relatively rare in clinical practice. The concept of MPMN was first proposed by Billroth et al. in 1889, and its clinical incidence rate is approximately 2% to 17% [4,5,6]. Generally classified according to the time interval between the onset of the two tumors, MPMNs are divided into synchronous MPMNs (with an interval of no more than six months) and metachronous MPMNs (with an interval longer than six months). At present, the pathogenesis of MPMNs has not been fully clarified. It is generally believed to be closely associated with age, autoimmune suppression or deficiency, environmental carcinogenic factors, iatrogenic factors such as radiotherapy and chemotherapy, and the carriage of tumor susceptibility genes [7].

The therapeutic principles for MPMNs are consistent with those for the first primary malignant tumor, aiming to achieve radical cure. For metachronous MPMNs, the treatment process usually involves sequential therapy for individual tumors. Relevant studies have demonstrated that metachronous MPMNs tend to have a more favorable prognosis than synchronous ones, and a longer interval between the two primary cancers correlates with a better prognosis.

Among breast cancer patients, about 4.2% to 17% may suffer from MPMNs, and this proportion has shown a gradual upward trend in recent years [8,9,10]. Multiple myeloma (MM) ranks as the second most common hematological malignancy in terms of incidence, predominantly affecting middle-aged and elderly people [11]. MM is mainly caused by the malignant proliferation of monoclonal plasma cells, with typical initial manifestations including hypercalcemia, renal impairment, anemia and bone pain [12]. With the continuous emergence of new drugs, the first-line treatment landscape for multiple myeloma is undergoing profound changes. The traditional triple-drug combination regimen consisting of proteasome inhibitors, immunomodulators and dexamethasone once served as the standard treatment. Nevertheless, this regimen often yields unsatisfactory efficacy in high-risk or relapsed and refractory patients, prompting the evolution of first-line treatment strategies from triple-drug combinations to four-drug combinations incorporating CD38 monoclonal antibodies [12,13,14]. Targeting the therapeutic goal of achieving deeper and more durable remission, four-drug regimens exhibit remarkable advantages and potential superior to triple-drug regimens. Multiple primary malignant neoplasms (MPMNs) frequently consist of combinations between breast cancer and other solid tumors, such as gynecologic, pulmonary and gastrointestinal malignancies. In contrast, metachronous multiple myeloma (MM) developing subsequent to breast invasive ductal carcinoma represents a rare clinical entity, which renders the present case distinctive from most reported MPMN pairs.

Numerous case reports have indicated overlapping diagnostic features between breast cancer and multiple myeloma [15,16,17]. Most patients documented in these reports presented typical manifestations of multiple myeloma such as hypercalcemia and osteolytic lesions. In contrast, the patient in this case maintained normal blood calcium levels without osteolytic changes throughout the diagnosis and treatment process, and mainly manifested myelosuppression symptoms including leukopenia, anemia and thrombocytopenia. Such clinical cases suggest that when patients develop new lesions that cannot be reasonably attributed to chemotherapy-related adverse reactions or conventional disease progression, clinicians need to broaden their differential diagnostic thinking and promptly conduct supplementary pathological examinations.

The patient in this case was pathologically diagnosed with invasive ductal carcinoma of the breast in February 2025, and was subsequently confirmed to have MM after an interval of 11 months. Given that the interval exceeded six months, the patient was diagnosed with metachronous MPMN. However, mild thrombocytopenia was already detected when breast cancer was first diagnosed, leaving uncertainties about whether multiple myeloma had already developed at that time and whether the thrombocytopenia was induced by multiple myeloma. Although cytotoxic chemotherapy and radiotherapy may elevate the risk of secondary hematologic malignancies, the timeline, disease features and clinical findings in this case do not support a causal link. The possibility of therapy-related multiple myeloma (t-MM) should nonetheless be considered. t-MM is uncommon and typically develops ≥5 years after genotoxic exposure, whereas MM was diagnosed merely 11 months following breast cancer identification. This short latent interval makes treatment-induced MM improbable. Notably, thrombocytopenia was present before anti-tumor therapy, suggestive of pre-existing hematologic abnormalities. Even so, we cannot exclude that antitumor treatment accelerated progression of undiagnosed subclinical MM. One limitation of this report is absent post-transfer follow-up data. Since subsequent care was delivered at an outside institution, details of MM treatment, serial hematologic surveillance and ongoing breast cancer status were unavailable.

Recent studies [18,19,20,21,22] have focused on the rare co-occurrence of breast cancer and multiple myeloma (MM), emphasizing prevalent diagnostic confusion between MM-induced osteolytic lesions and breast cancer bone metastasis. Multiple clinical cases demonstrated that bone lesions in breast cancer survivors are frequently misinterpreted as metastatic recurrence, whereas comprehensive laboratory screening including serum protein electrophoresis, free light chain detection and bone marrow biopsy can effectively confirm a secondary MM. Such dual malignancies arise in both female and rare male breast cancer patients, possibly attributable to treatment-related immune dysregulation and shared genetic susceptibility. Notably, ixazomib-based regimens have exhibited promising anti-tumor activity against hormone receptor-positive breast cancer, achieving complete pathological response in patients with concurrent MM, indicating the potential value of proteasome inhibitors in solid tumor treatment. Moreover, synchronous autoimmune disorders such as systemic lupus erythematosus may further complicate the clinical course after breast cancer therapy. Given that current evidence is predominantly limited to case reports, large-scale and prospective studies are still required to establish standardized diagnostic and therapeutic strategies for patients with overlapping breast cancer and multiple myeloma.

With the advancement of cancer screening and diagnostic and therapeutic technologies, the incidence of MPMNs is on the rise. Each tumor involved in MPMNs possesses independent pathological characteristics, therapeutic demands and biological behaviors, and such complexity sets higher standards for clinical diagnosis and treatment. We consider comprehensive genomic profiling (CGP) worthy of consideration for this patient. For patients with metachronous MPMN, CGP can help identify shared or unique genomic alterations between the two malignancies, clarify potential clonal relationships, and guide individualized targeted therapy. Nevertheless, we would not routinely mandate CGP at present. The implementation of CGP is constrained by economic costs, tissue availability, and local clinical accessibility. In our current clinical setting, CGP is not a standard test for patients with double primary breast cancer and multiple myeloma. If the patient develops disease progression or requires subsequent systemic treatment in the future, we will actively recommend CGP to explore actionable molecular targets.

The co-occurrence of breast cancer and multiple myeloma constitutes a relatively rare combination of multiple primary malignant tumors in clinical settings. Although there is no direct etiological correlation between the two diseases, their development may be linked to common predisposing factors such as advanced age, genetic susceptibility and shared environmental exposure. In some cases, long-term secondary effects resulting from breast cancer treatment should also be taken into consideration.

In clinical practice, overlapping clinical manifestations including bone pain and anemia as well as imaging features such as osteolytic bone destruction often lead to confusion with breast cancer bone metastasis. In medical institutions with limited diagnostic and therapeutic resources or insufficient clinical experience, clinicians who confine their diagnostic thinking to a single primary cancer and fail to arrange targeted examinations in a timely manner face risks of missed diagnosis or delayed diagnosis. Therefore, clinicians, especially grassroots medical practitioners, should strengthen the awareness of screening for multiple primary malignant tumors.

This study has several limitations. First, as a single case report, firm conclusions regarding the epidemiological association and shared molecular mechanisms between breast cancer and multiple myeloma cannot be drawn. Second, germline genetic testing and serial comprehensive genomic profiling were not performed, which restricts further exploration of potential predisposing factors and clonal relationships between the two malignancies. In addition, we cannot definitively confirm whether mild thrombocytopenia at breast cancer diagnosis originated from subclinical multiple myeloma in the absence of earlier bone marrow evaluation. Prospective larger cohort investigations are needed to address these questions.

For patients with breast cancer, the possibility of secondary primary cancers such as multiple myeloma should be highly suspected if they develop unexplained bone pain, anemia, hypercalcemia or renal dysfunction that cannot be explained by tumor metastasis or conventional complications during postoperative recovery or follow-up visits. Timely testing with serum protein electrophoresis, immunofixation electrophoresis and bone marrow biopsy is recommended for breast cancer patients presenting with unexplained cytopenia or bone pain. These examinations are not standard surveillance measures in breast cancer guidelines and should only be performed in patients with clinical suspicion of concurrent plasma cell disease. A multidisciplinary team (MDT) strategy to deliver individualized care may improve long-term patient outcomes.

## 4. Conclusions

Multiple primary malignant neoplasms (MPMNs) are rare clinical entities with an incidence of 2–17%, and the concurrent presentation of breast cancer and multiple myeloma (MM) represents an uncommon subtype associated with significant diagnostic challenges due to overlapping clinical and imaging features. Overlapping symptoms often lead to misdiagnosis as bone metastasis, necessitating targeted examinations such as serum protein electrophoresis and bone marrow biopsy for accurate diagnosis. Shared risk factors including age and genetic susceptibility may contribute to disease co-occurrence, while proteasome inhibitors show therapeutic potential and comprehensive genomic profiling may guide personalized treatment despite current limitations in routine application. As a single case report, this study cannot establish definitive epidemiological or molecular associations, highlighting the need for large-scale prospective research to develop standardized diagnostic and therapeutic strategies. Clinicians should enhance MPMN screening awareness, implement multidisciplinary approaches, and conduct targeted evaluations in breast cancer patients with unexplained symptoms to improve diagnostic accuracy and patient outcomes.

## Data Availability

The datasets used during the current study are available from the corresponding author on reasonable request.

## Supplementary Materials

The following supporting information can be downloaded at https://www.mdpi.com/article/10.3390/curroncol33080473/s1, Table S1: Dynamic Hematological Parameters During Neoadjuvant Chemoradiotherapy.

## Abbreviations

ALT	Alanine transaminase
AML	Acute myeloid leukemia
ANA	Antinuclear antibody
BI-RADS	Breast Imaging Reporting and Data System
CA125	Carbohydrate antigen 125
CA15-3	Carbohydrate antigen 15-3
CEA	Carcinoembryonic antigen
CGP	Comprehensive genomic profiling
CT	Computed tomography
CD	Cluster of differentiation
ECOG	Eastern Cooperative Oncology Group
eGFR	Estimated glomerular filtration rate
ENA	Extractable nuclear antigen
ESBLs	Extended-spectrum beta-lactamases
GLOBOCAN	Global Cancer Observatory
HGB	Hemoglobin
HER-2	Human epidermal growth factor receptor 2
HPF	High-power fields
IHC	Immunohistochemistry
IgA	Immunoglobulin A
IgG	Immunoglobulin G
IgM	Immunoglobulin M
MCV	Mean corpuscular volume
MDS	Myelodysplastic syndrome
MDT	Multidisciplinary team
MP	Miller–Payne
MPMN	Multiple primary malignant neoplasms
MM	Multiple myeloma
PLT	Platelet
PR	Partial response; Progesterone receptor
PTV	Planning target volume
PTVtb	Tumor bed planning target volume
t-MM	Therapy-related multiple myeloma
TCbHP	Docetaxel + Carboplatin + Pertuzumab + Trastuzumab
THP	Docetaxel + Pertuzumab + Trastuzumab
WBC	White blood cell
